# Expression Pattern and Biochemical Properties of Zebrafish N-Acetylglutamate Synthase

**DOI:** 10.1371/journal.pone.0085597

**Published:** 2014-01-22

**Authors:** Ljubica Caldovic, Nantaporn Haskins, Amy Mumo, Himani Majumdar, Mary Pinter, Mendel Tuchman, Alison Krufka

**Affiliations:** 1 Center for Genetic Medicine Research, Children's National Medical Center, Washington D.C., United States of America; 2 Department of Integrative Systems Biology, The George Washington University, Washington D.C., United States of America; 3 Molecular and Cellular Biology Program, University of Maryland, College Park, Maryland, United States of America; 4 American Society for Radiation Oncology, Fairfax, Virginia, United States of America; 5 Department of Biological Sciences, Rowan University, Glassboro, New Jersey, United States of America; University of Cantebury, New Zealand

## Abstract

The urea cycle converts ammonia, a waste product of protein catabolism, into urea. Because fish dispose ammonia directly into water, the role of the urea cycle in fish remains unknown. Six enzymes, N-acetylglutamate synthase (NAGS), carbamylphosphate synthetase III, ornithine transcarbamylase, argininosuccinate synthase, argininosuccinate lyase and arginase 1, and two membrane transporters, ornithine transporter and aralar, comprise the urea cycle. The genes for all six enzymes and both transporters are present in the zebrafish genome. NAGS (EC 2.3.1.1) catalyzes the formation of N-acetylglutamate from glutamate and acetyl coenzyme A and in zebrafish is partially inhibited by L-arginine. NAGS and other urea cycle genes are highly expressed during the first four days of zebrafish development. Sequence alignment of NAGS proteins from six fish species revealed three regions of sequence conservation: the mitochondrial targeting signal (MTS) at the N-terminus, followed by the variable and conserved segments. Removal of the MTS yields mature zebrafish NAGS (zfNAGS-M) while removal of the variable segment from zfNAGS-M results in conserved NAGS (zfNAGS-C). Both zfNAGS-M and zfNAGS-C are tetramers in the absence of L-arginine; addition of L-arginine decreased partition coefficients of both proteins. The zfNAGS-C unfolds over a broader temperature range and has higher specific activity than zfNAGS-M. In the presence of L-arginine the apparent V_max_ of zfNAGS-M and zfNAGS-C decreased, their K_m_^app^ for acetyl coenzyme A increased while the K_m_^app^ for glutamate remained unchanged. The expression pattern of NAGS and other urea cycle genes in developing zebrafish suggests that they may have a role in citrulline and/or arginine biosynthesis during the first day of development and in ammonia detoxification thereafter. Biophysical and biochemical properties of zebrafish NAGS suggest that the variable segment may stabilize a tetrameric state of zfNAGS-M and that under physiological conditions zebrafish NAGS catalyzes formation of N-acetylglutamate at the maximal rate.

## Introduction

Ammonia is an obligatory waste product of protein catabolism that is highly toxic to the brain [1]. Fish and other aquatic animals excrete ammonia directly into water, while most land animals use either the urea cycle or the uric acid pathway to convert neurotoxic ammonia into non-toxic urea or uric acid, which are easily excreted [2]. Although adult fish excrete ammonia directly into water, urea cycle enzymes have been found in 23 species of fish [3]. The genomes of zebrafish, pufferfish (*Fugu rubripes*), freshwater pufferfish (*Tetraodon nigroviridis*) and African coelacanth (*Latimeria chalumnae*) encode enzymes and transporters needed for the production of urea from nitrogenous waste [3], [4]. Many fish are capable of ureagenesis and there are several fish species for which a need for the urea cycle can be explained. Lungfish are periodically exposed to air and use the urea cycle to dispose of ammonia during periods of water shortage [5]–[8]. Sharks, skates and rays use urea as an osmolyte [9]–[14]. The urea cycle detoxifies ammonia in the fish that live in alkaline water and cannot excrete ammonia through the gills [15]–[20]. Since most fish rarely encounter water with high ammonia concentration [21], the need for ureagenesis in zebrafish and other fish is not clear. In these fish the urea cycle may be important for embryonic development. Zebrafish (*Danio rerio*), Atlantic cod (*Gadus morhua*) and rainbow trout (*Oncorhynchus mykiss*) embryos excrete most of their nitrogen waste as urea [22]–[25]. Indeed, mRNA and activities of several urea cycle enzymes were present in developing rainbow trout, Atlantic cod, Atlantic halibut (*Hipoglossus hipoglossus*), walking catfish (*Clarias batrachus*), pacu (*Piaractus mesopotamicus*) and zebrafish early in development [22], [23], [26]–[30]. However, expression of all enzymes and transporters required for the function of urea cycle were not measured in these studies.

Five enzymes of the urea cycle catalyze conversion of ammonia into urea. In addition, N-acetylglutamate synthase (NAGS; EC 2.3.1.1), ornithine/citrulline transporter (ORNT) and aspartate/glutamate transporter (also known as either Aralar2 or citrin) are required for the normal function of the urea cycle in mammals [1]. The first reaction of the urea cycle is the formation of carbamylphosphate (CP). In mammals, carbamylphosphate synthetase I (CPS1) produces CP from ammonia, bicarbonate and ATP [1]. In fish, the formation of CP is catalyzed by carbamylphosphate synthetase III (CPS3), with bicarbonate, ATP and either glutamine or ammonia as substrates [9], [30], [31]. Ornithine transcarbamylase (OTC; EC 2.1.3.3), the next enzyme in the pathway, catalyzes the formation of citrulline from CP and ornithine [1]. ORNT transports citrulline into the cytoplasm, where it is converted into urea and ornithine by argininosuccinate synthase (ASS; EC 6.3.4.5), argininosuccinate lyase (ASL; EC 4.3.2.1) and arginase 1 (Arg1; EC 3.5.3.1) [1]. Urea is excreted and ornithine is transported into mitochondria by ORNT for another turn of the urea cycle [1].

N-acetylglutamate (NAG), which is formed enzymatically by NAGS from glutamate and acetylcoenzyme A (AcCoA) is an essential allosteric activator of CPS1; NAG deficiency results in a block of ureagenesis [32], [33]. NAG also activates CPS3, but the effect of NAG on CPS3 activity varies in different fish species. In Atlantic halibut, spiny dogfish (*Squalus acanthias*) and largemouth bass (*Micropterus salmoides*) NAG is required for enzymatic activity of CPS3 at low glutamine concentrations [9], [30], [31], [34], but partially purified CPS3 from the Lake Magadi tilapia (*Oreochromis alcalicus*) remains active without NAG [16]. NAG has been found in the liver of adult spiny dogfish, largemouth bass, rainbow trout and gulf toadfish (*Opsanus beta*), as well as in the muscles of adult rainbow trout and gulf toadfish [35] suggesting that NAGS is expressed in these tissues.

L-arginine is an allosteric regulator of NAGS [3], [32]. Microbial and plant NAGS, which catalyze formation of NAG as the first intermediate in arginine biosynthesis, are inhibited by L-arginine, mammalian NAGS is activated by L-arginine whereas fish NAGS is partially inhibited by L-arginine [3], [36]. Therefore fish NAGS appears to be an intermediate form on the evolutionary path from microbial to mammalian NAGS. Experiments with partially purified rat and *E. coli* NAGS have shown that L-arginine also affects the oligomerization state of these two enzymes [37], [38], while the oligomerization state of purified recombinant NAGS from *Neisseria gonorrhoeae* and *Pseudomonas aeruginosa*, which are similar to *E. coli* NAGS, and vertebrate-like N-acetylglutamate synthase/kinase from *Maricaulis maris* do not change in the presence of L-arginine [39]–[42]. This diversity of biochemical and biophysical properties of NAGS from different organisms may be related to the changing role of NAG through evolution [43].

To aid in the understanding of NAGS evolution, we determined the biochemical and biophysical properties of zebrafish NAGS, and the effect of L-arginine on the oligomerization state and catalytic properties of the enzyme. We also examined the expression pattern of NAGS and all other urea cycle enzymes and transporters in developing zebrafish to determine whether it can explain ureotely in early zebrafish embryos.

## Results and Discussion

### Expression of Urea Cycle Genes During Zebrafish Development

**Figure 1:**
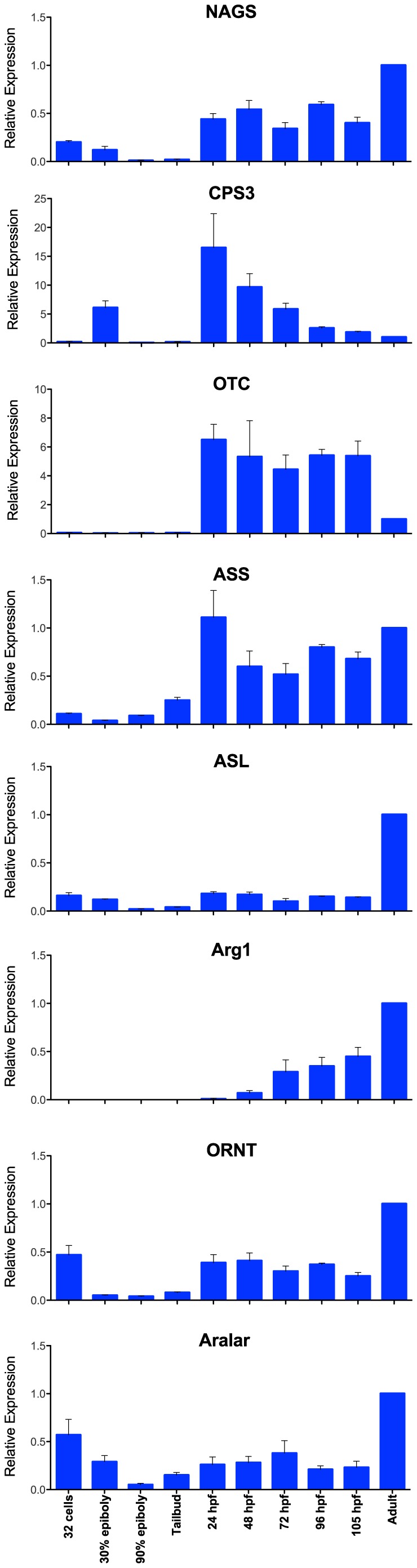
Relative expression of urea cycle genes in developing zebrafish. mRNA levels were measured at nine developmental stages: 32 cells, 30% epiboly (4.6 hpf), 90% epiboly (9 hpf), tailbud (10 hpf), 24 hpf, 48 hpf, 72 hpf, 96 hpf, 105 hpf, and normalized to the abundance of each mRNA in adult zebrafish. The scales of y-axes differ due to different expression patterns of zebrafish urea cycle genes.

The mRNA expression pattern of NAGS and other urea cycle genes during zebrafish development was determined using quantitative RT-PCR. RNA from the adult fish was used as a reference. The eight genes of the urea cycle have three distinct patterns of expression in developing zebrafish (Figure 1). NAGS, ASS, ASL, ORNT and citrin are expressed in the 32-cell embryos, suggesting that these mRNA are maternal. These five genes are also expressed in the late-blastula stage; their expression is low during gastrulation and appears to increase at the tailbud stage and during the first four days of development (Figure 1). Relative expression of the NAGS, ASS, ASL, ORNT and Aralar1 is similar or higher in adult fish than during development. The high relative expression of ASS, ASL, ORNT and Aralar1 in adulthood is likely related to their function in processes other than the urea cycle. Interestingly, the relative expression of NAGS is higher in adult zebrafish than in embryos suggesting that NAGS could also have a role in zebrafish physiology beyond ureagenesis. Expression of CPS3 and OTC is higher during embryogenesis than in adult zebrafish (Figure 1). Since the only known functions of CPS3 and OTC in fish are in urea and arginine biosynthesis [44], [45] their expression pattern is consistent with high rate of ureagenesis during embryonic and larval development [25], [26]. The relative expression of Arg1 begins to increase after the first day of development and continues to increase thereafter and, as a result, is higher in adult than in developing zebrafish (Figure 1). A high relative expression of Arg1 in adult zebrafish may be related to a possible role of this enzyme in arginine catabolism. Alternatively, the function of Arg1 could be to catalyze the formation of ornithine, which could then be used as a precursor of polyamine biosynthesis [46].

*In situ* hybridization has been used in other studies to determine the tissue distribution of CPS3, OTC, ASS and ASL mRNA at different developmental stages. At 32 hpf all four genes were expressed in the embryonic endoderm [26]. Expression data for ASS and ASL are also available in the curated collection of gene expression data in zebrafish [47], [48]. Late in the blastula stage (30% epiboly) and during early gastrulation (50% epiboly) ASS is expressed in the deep cell layer and the forerunner cell group, respectively [48]. These two cell types later give rise to mesodermal tissues in the tail [49]. ASS is expressed in the endoderm at 5–9 and 14–19 somite stages (10 and 16 hpf, respectively) [48]. The expression pattern of ASL is available for three developmental stages. At 19–24 hpf, which corresponds to 20–25 somites and Prim-5 developmental stages, ASL mRNA is expressed in the pronephric duct and solid lens vesicle [47]. Later, at 24–30 hpf and 42–48 hpf, ASL mRNA is expressed in the lens and pronephric duct [47], which is different from the ASL expression pattern observed by LeMoine and Walsh [26]. Expression of ASL mRNA in the zebrafish lens suggests that ASL may have similar function in the lens of fish and birds, where ASL functions as δ-crystalin [50]–[56] while expression in the pronephric duct suggests that ASL could be involved in renal arginine biosynthesis, similar to ASL function in mammals [57].

Expression of all eight urea cycle genes between the 24 and 105 hpf stages coincides with neurogenesis [49]. During this period gills are forming [58] and developing zebrafish are transitioning from ureotely to ammonotely [25]. Fish embryos and larvae rely on protein and amino acids from the yolk sac to synthesize cellular proteins needed for growth and development as well as fuel embryogenesis and larval development before the onset of feeding [59]–[63]. The use of amino acids for ATP synthesis results in the production of ammonia [64]. This ammonia may not easily diffuse out of fish embryos and larvae because direct contact with water and fully formed gills are lacking [65], [66], and would accumulate in the fish embryos and larvae, as has been observed in developing zebrafish [26]. Elevated ammonia could damage tissues, especially the developing brain, if not converted into urea by the urea cycle.

Developing zebrafish embryos appear to be ureotelic during the first 48 hrs of development as they excrete between 40 and 80% of nitrogen waste as urea in that period [25], [67]. Between 48 and 72 hpf zebrafish become ammonotelic as they begin to excrete ammonia via ionophore cells that express ammonia transporter *Rhcg1* [67]–[69]. Our results and an earlier study of CPS3, OTC, ASS and ASL expression pattern [26] can explain production of urea after the first day of zebrafish development. However, the physiological process responsible for the excretion of urea during the first 24 hpf remains to be elucidated as OTC and Arg1, both required for urea production, are not expressed in developing zebrafish during this time. One possibility is that arginase-2, which is a mitochondrial enzyme that catalyzes the same reaction as Arg1, could enable urea production in developing zebrafish embryos before onset of expression of Arg-1. Arginase-2 is expressed in the axial mesoderm and forerunner cells during gastrulation; later in development arginase-2 mRNA is expressed in the central nervous system and in the mucus secreting cells, which are part of the immune system [47]. Therefore, only a complicated transport of metabolites between different cell types and tissues could account for urea production before the onset of Arg1 expression.

Unlike mammals, which have two aspartate/glutamate transporters Aralar1 and citrin, the zebrafish genome harbors only one gene, annotated as Aralar1, with similarity to mammalian aspartate/glutamate transporters. The protein sequence of zebrafish Aralar1 is 78% and 75% identical to human Aralar1 and citrin, respectively, whereas human Aralar1 and citrin sequences are 77% identical. Zebrafish Aralar was included in expression analysis because of its role in mammalian ureagenesis [70] but additional studies are needed to elucidate the role of Aralar in fish physiology.

Our results show that five enzymes of the urea cycle, NAGS, CPS3, OTC, ASS and ASL, and two transporters are all expressed between 24 and 48 hpf. This expression pattern is also consistent with the function of these enzymes in citrulline and/or arginine biosynthesis. Onset of expression of Arg1 after hatching is not unique to zebrafish and has also been observed in pacu, rainbow trout and Atlantic cod [22], [23], [29], suggesting that urea cycle enzymes may have different physiological roles at different developmental stages. Additional experiments are needed to identify precisely which cell types harbor urea cycle enzymes at different developmental stages and to draw conclusions about functions of the urea cycle in fish.

### Domain Structure of Zebrafish NAGS

**Figure 2:**
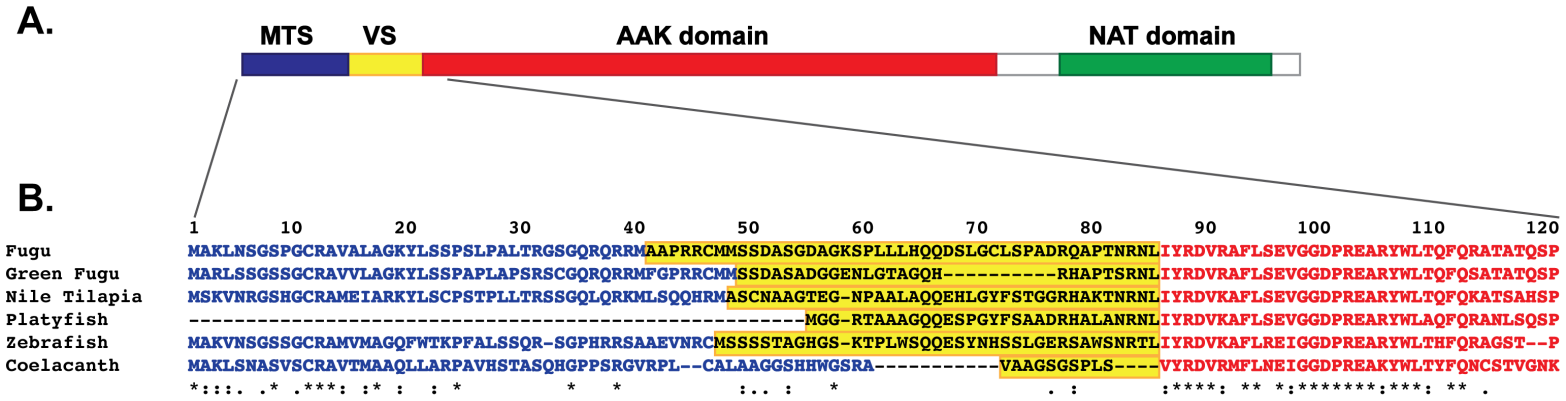
Sequence conservation and domain structure of fish NAGS proteins. **A.** Domain structure of fish NAGS. MTS – mitochondrial targeting signal shown in blue; VS – variable segment shown in yellow; AAK – amino acid kinase domain shown in red; NAT – N-acetyltransferase domain shown in green. **B.** Sequence alignment of the N-terminal region of six fish NAGS proteins. Predicted MTS are shown in blue typeface. The variable segment is highlighted in yellow. The first 33–35 amino acids of the AAK domain are shown in red typeface.

Mammalian NAGS proteins consist of three segments with different degrees of sequence conservation, the mitochondrial targeting sequence (MTS), the variable segment and the conserved segment [71], [72]. The NAGS genes and corresponding proteins were identified in genomes of Nile tilapia (*Oreochromis niloticus*), coelacanth [4] and platyfish (*Xiphophorus maculates*) and analyzed with previously identified NAGS from zebrafish, pufferfish and freshwater pufferfish [3]. Like mammalian NAGS, alignment of fish NAGS revealed three regions of conservation: the MTS at the N-terminus followed by the variable segment and the conserved segment, which comprises the amino acid kinase (AAK) and N-acetyltransferase (NAT) structural domains (Figure 2A). The MTS in fish NAGS is 40–58 amino acids long with approximately 50% conservation (Figure 2B). The MTS appears to be absent from the platyfish NAGS either because it is not imported into the mitochondria or the corresponding sequence may be missing from the current platyfish genome assembly. The MTS is presumably removed upon import in the mitochondria, resulting in the mature NAGS (NAGS-M). The variable segment of fish NAGS proteins are poorly conserved and are between 10 and 45 amino acids long, which is shorter than the variable segment of mammalian NAGS [71], [72] (Figure 2B). Within the conserved segment of fish NAGS (NAGS-C), the NAT domain has a higher degree of conservation (62% identical amino acids) than the AAK domain (38% identity). The N-terminus of zfNAGS-M protein was chosen based on the prediction of MTS by the MitoProt software package [73] while the N-terminus of zfNAGS-C was determined based on the alignment of zebrafish and mammalian NAGS.

### Biochemical Properties of Zebrafish NAGS

**Figure 3:**
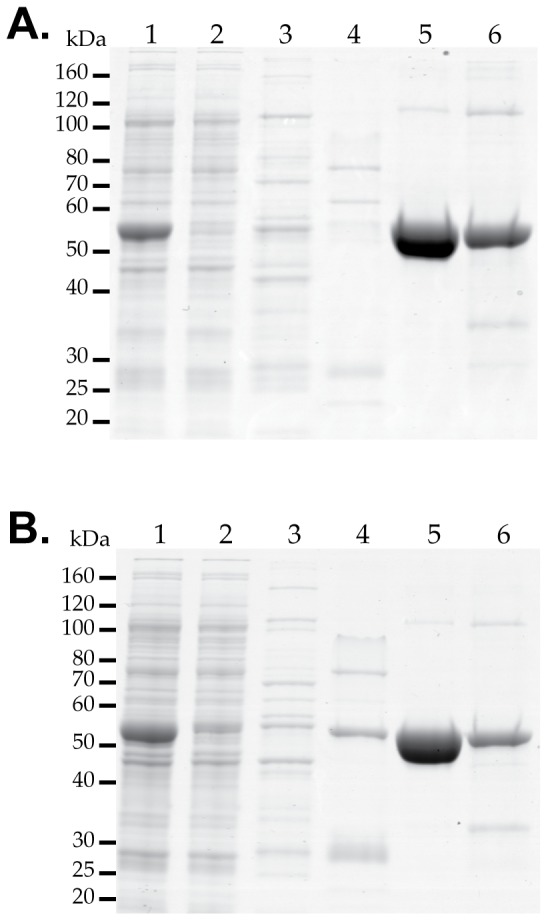
Purification of recombinant zfNAGS-M and zfNAGS-C. The zfNAGS-M (**A**) and zfNAGS-C (**B**) with the N-terminal polyhistidine tag were overexpressed in *E. coli* and purified using nickel-affinity column. Lane 1 – cell lysate; lane 2 – flow-through fraction; lane 3 – wash fraction; lane 4 – elution with 125 mM imidazole; lane 5 – elution with 250 mM imidazole; lane 6 – elution with 500 mM imidazole.

Zebrafish NAGS, which is partially inhibited by L-arginine, is an intermediary on the evolutionary path of changing the allosteric effect of arginine on NAGS from inhibition in microbes and plants to activation in mammals [3]. The zfNAGS-M and zfNAGS-C were overexpressed in *E. coli* and purified to homogeneity (Figure 3). Denatured zfNAGS-M and zfNAGS-C migrated as single bands of approximately 55 and 52 kDa, respectively (Figure 3). This is in good agreement with the predicted molecular weights of 55,498 and 52,752 Da for the zfNAGS-M and zfNAGS-C, respectively.

**Figure 4:**
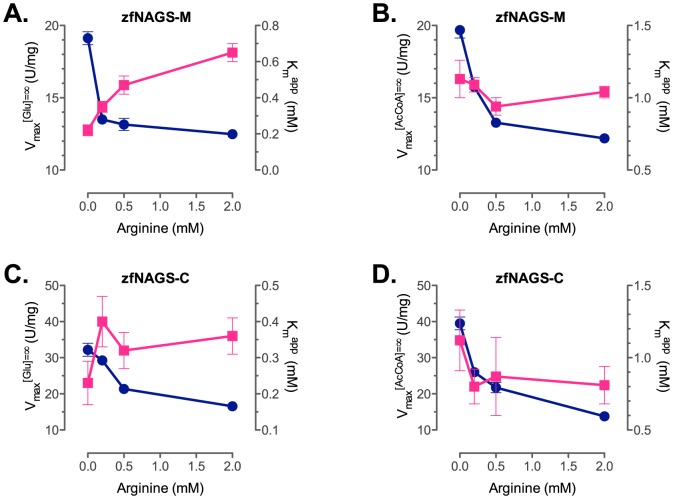
Effect of L-arginine on biochemical properties of zfNAGS-M and zfNAGS-C. K_m_^app^ and apparent V_max_ for AcCoA (A and C) and glutamate (B and D) when increasing amounts of arginine were added to zfNAGS-M and zfNAGS–C. (Blue – V_max_; Magenta – K_m_^app^). Error bars represent standard errors of the fitting parameters for the Michaelis-Menten equation.

Purified zfNAGS-M and zfNAGS-C were used to measure enzymatic activities at variable concentrations of one of the substrates while fixing the other substrate at a high concentration to determine apparent maximal velocity (V_max_) and K_m_^app^ for AcCoA and glutamate (Figure 4 and Figure S1, 1). The apparent V_max_ and K_m_^app^ were determined in the presence of varying concentrations of arginine (Figure 4 and Figure S1, Table 1). The apparent V_max_ of zfNAGS-C was approximately double the V_max_ of zfNAGS-M (Table 1 and Figure 4). This effect of removing the variable segment on enzymatic activity of zebrafish NAGS is similar to the effect of removing the variable segment of mouse and human NAGS [74]. The apparent V_max_ of zfNAGS-M and zfNAGS-C in the absence of arginine (Table 1) were comparable to the corresponding V_max_ of mouse NAGS [74]. The K_m_^app^ for AcCoA and glutamate of both proteins (Table 1) were three- to four-fold lower than corresponding K_m_^app^ of mammalian NAGS [74]. The intramitochondrial concentrations of AcCoA and glutamate in fish are not known, but if they are similar to intramitochondrial concentrations of NAGS substrates in rat hepatocytes (0.6–2.7 mM for AcCoA [75] and 3–15 mM for glutamate [76]) that would suggest that zebrafish NAGS catalyzes formation of NAG at close to the maximal rate.

**Table 1:** Comparison of biochemical properties of purified zebrafish and mouse NAGS proteins.

Protein	L-Arginine Concentration	AcCoA		Glutamate	
		V_max_ (U/mg)	K_m_^app^ (mM)	V_max_ (U/mg)	K_m_^app^ (mM)
zfNAGS-M	0 mM	19.12±0.45^a^	0.22±0.03^a^	19.69±0.56^a^	1.13±0.13^a^
	0.2 mM	13.50±0.29	0.35±0.03	15.75±0.17	1.09±0.05
	0.5 mM	13.15±0.42	0.47±0.05	13.27±0.18	0.94±0.06
	2.0 mM	12.48±0.33	0.65±0.05	12.19±0.10	1.04±0.04
zfNAGS-C	0 mM	32.20±1.8	0.23±0.06	39.51±1.78	1.12±0.21
	0.2 mM	29.25±1.20	0.40±0.07	26.00±0.78	0.80±0.12
	0.5 mM	21.34±0.87	0.32±0.05	21.73±1.43	0.87±0.27
	2.0 mM	16.54±0.60	0.36±0.05	13.77±0.45	0.81±0.13
mNAGS-M^b^	0 mM	23.90±0.74	1.01±0.09	23.15±0.93	2.91±0.19
mNAGS-C^b^	0 mM	37.20±1.19	1.03±0.09	38.29±0.42	3.02±0.10

a Values represent fitting parameters to Michaelis-Menten equation and the associated standard errors.

b Values for mouse NAGS-M and NAGS-C are from Table 2 in Caldovic et al. [74].

Addition of L-arginine to zfNAGS-M and zfNAGS-C resulted in a reduction of apparent V_max_ of both proteins by approximately 30 and 50%, respectively (Figure 4 and Table 1). The effect of L-arginine on K_m_^app^ differed for AcCoA and glutamate. The K_m_^app^ of both proteins for AcCoA increased in the presence of L-arginine while the K_m_^app^ for glutamate did not change (Figure 4 and Table 1). Changes in both K_m_^app^ and apparent V_max_ in the presence of L-arginine and binding of arginine to both zfNAGS-M and zfNAGS-C in the absence of substrates suggests a hyperbolic mode of inhibition; for both proteins the concentration of arginine that has half-maximal effect on the apparent V_max_ and K_m_^app^ does not exceed 0.65 mM. The intramitochondrial concentration of arginine in fish is not known, but if it is similar to the L-arginine levels in mammalian mitochondria (0.12–1.34 mM [77], [78]), the effect of L-arginine *in vivo* would be at most a 30% reduction in the rate of NAG synthesis. The low K_m_^app^ for AcCoA and glutamate relative to intramitochondrial concentrations of these metabolites and likely low level of inhibition of zebrafish NAGS by L-arginine suggest that substrate and cofactor concentrations likely do not control the production of NAG in developing zebrafish, a situation different from the regulatory role of NAG in mammals [38], [79].

### Oligomerization State of Zebrafish NAGS

**Figure 5:**
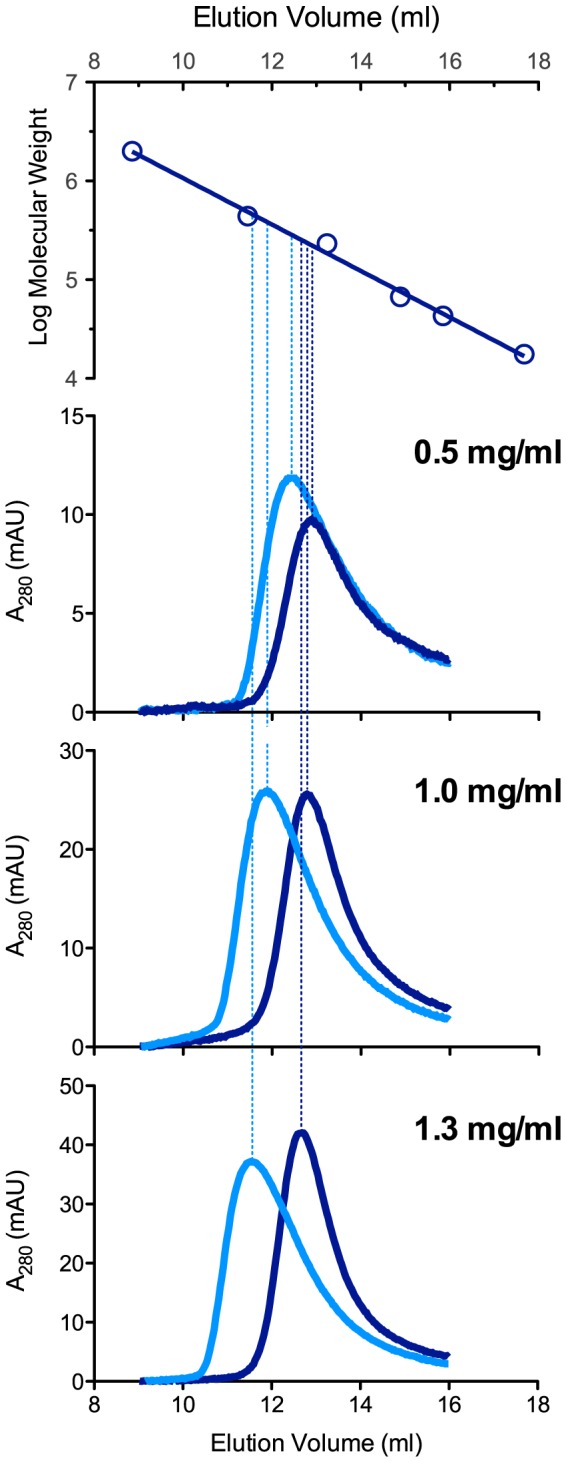
Analytical gel chromatography of zfNAGS-M with and without L-arginine. The top panel shows a semi-logarithmic plot of molecular mass vs. elution volume. Open circles correspond to elution volumes of blue dextran (2000 kDa), ferritin (440 kDa), catalase (232 kDa), aldolase (158 kDa), bovine serum albumin (66 kDa), ovalbumin (43 kDa), and myoglobin (16 kDa). Lower panels show absorption at 280 nm as a function of elution volume. Concentration of zfNAGS-M loaded on the column is indicated in each panel. Dark blue – elution profiles of zfNAGS-M without arginine. Cyan – elution profiles of zfNAGS-M in the presence of 1 mM L-arginine.

The oligomerization state of zebrafish NAGS was investigated because previous studies have shown that oligomerization of partially purified NAGS from *E. coli* and rat changes in the presence of L-arginine [37], [38]. On the other hand, purified recombinant NAGS from *N. gonorrhoeae* and *P. aeruginosa*, which are similar to NAGS from *E. coli*, were stable hexamers in the presence or absence of L-arginine [39], [40], [42]. We used analytical gel chromatography to investigate oligomerization state of zebrafish NAGS in solution. In the absence of L-arginine elution volume of zfNAGS-M decreases by 0.2 ml as concentration of loaded protein increased (Figure 5 and Table 2). In the range of tested concentrations (0.5–1.3 mg/ml) elution volumes of zfNAGS-M correspond to the molecular weight of 234±12 kDa. Since the molecular weight of zfNAGS-M, calculated based on its amino acid sequence, is 55.5 kDa, the zfNAGS-M appears to be a tetramer in solution. This is not surprising as crystal structures of bifunctional NAGS-K from *Maricaulis maris* [41] and yeast N-acetylglutamate kinase (NAGK) [80] revealed a tetrameric structure of these two proteins, which are evolutionarily related to zfNAGS [3]. In addition to a slight decrease of elution volume with increasing concentration of zfNAGS-M, the elution profiles of zfNAGS-M in the absence of L-arginine were not symmetrical. These behaviors of zfNAGS-M are consistent with an ensemble of oligomers that equilibrate rapidly compared to their retention time or elution volume. Moreover, the tetrameric oligomerization state appears to predominate at all tested concentrations of zfNAGS-M in the absence of L-arginine. Alternatively, asymmetric elution profiles of zfNAGS-M could indicate that it interacts with the column stationary phase. In the presence of 1 mM L-arginine the elution volume of zfNAGS-M markedly decreased and was dependent on the concentration of the protein (Figure 5 and Table 2). This and the asymmetric elution profiles of zfNAGS-M in the presence of 1 mM L-arginine suggest a distribution of oligomerization states upon binding of L-arginine.

**Table 2:** Molecular weights and elution volumes of zfNAGS-M and zfNAGS-C in the presence and absence of L-arginine.

Protein	Concentration	Molecular Weight (kDa)		Elution volume (ml)	
		no L-Arg	1 mM L-Arg	no L-Arg	1 mM L-Arg
zfNAGS-M	0.5 mg/ml	222	292	12.89	12.41
	1.0 mg/ml	233	398	12.79	11.89
	1.3 mg/ml	247	492	12.69	11.53
zfNAGS-C	0.5 mg/ml	252	355	12.67	12.06
	1.0 mg/ml	258	483	12.63	11.53
	1.5 mg/ml	283	–a	12.47	10.81
	2.0 mg/ml	282	–a	12.48	10.58

a Elution volume was between elution volumes of the ferritin and blue dextran calibration standards.

**Figure 6:**
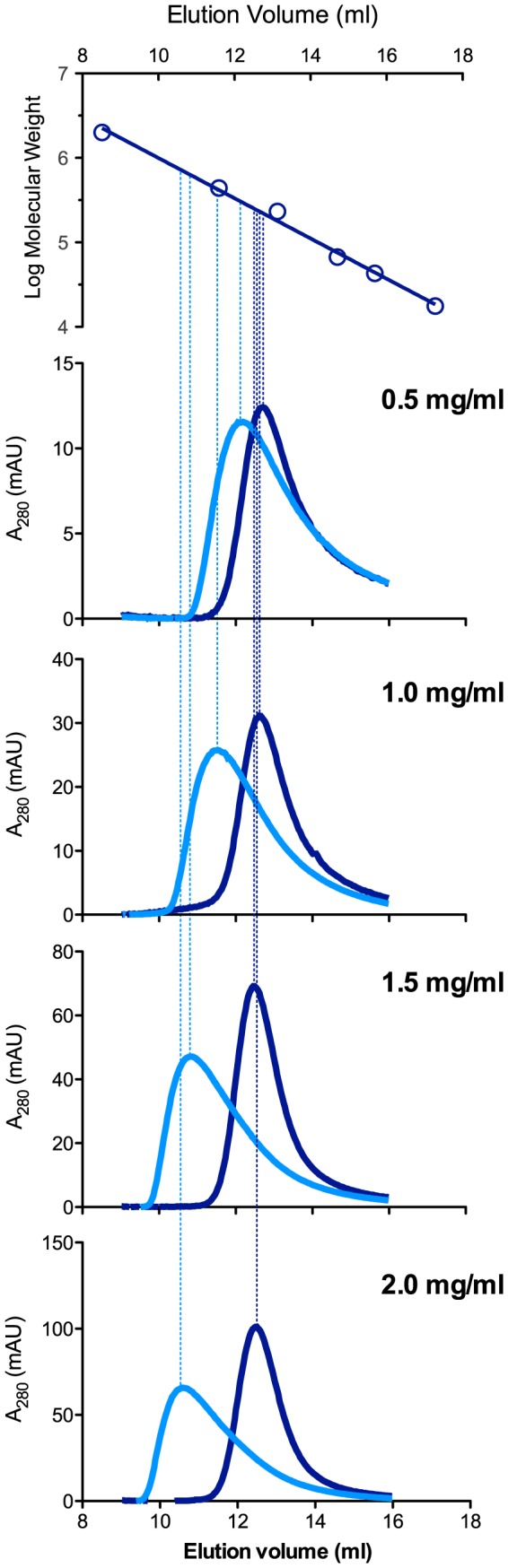
Analytical gel chromatography of zfNAGS-C with and without L-arginine. The top panel shows a semi-logarithmic plot of molecular mass vs. elution volume. Open circles correspond to elution volumes of blue dextran (2000 kDa), ferritin (440 kDa), catalase (232 kDa), aldolase (158 kDa), bovine serum albumin (66 kDa), ovalbumin (43 kDa), and myoglobin (16 kDa). Lower panels show absorption at 280 nm as a function of elution volume. The concentration of zfNAGS-C loaded on the column is indicated in each panel. Dark blue - elution profiles of zfNAGS-C without L-arginine. Cyan – elution profiles of zfNAGS-C in the presence of 1 mM L-arginine.

The elution peaks of zfNAGS-C were asymmetric at all protein concentrations and in the presence and absence of L-arginine (Figure 6) suggesting that this protein is an ensemble of oligomers that are rapidly equilibrating and cannot be resolved under conditions used in this experiment. The elution volumes of zfNAGS-C in the absence of L-arginine corresponded to a molecular weight of 269±16 kDa. Because the calculated molecular weight of zfNAGS-C monomer is 52.7 kDa, the experimental 269 kDa corresponds to pentameric oligomerization state of zfNAGS-C in solution, which is unlikely because NAGS and vertebrate-like NAGK with known three-dimensional structures are either hexamers or tetramers [41], [42], [80]. It is more likely that the elution volume of zfNAGS-C reflects average hydrodynamic properties of the ensemble of oligomers in rapid exchange. The elution volume of zfNAGS-C decreased and was dependent on its concentration in the presence of L-arginine (Figure 6 and Table 2) suggesting a shift in the ensemble of oligomers towards higher oligomerization states.

### Thermal Unfolding of Zebrafish NAGS

**Figure 7:**
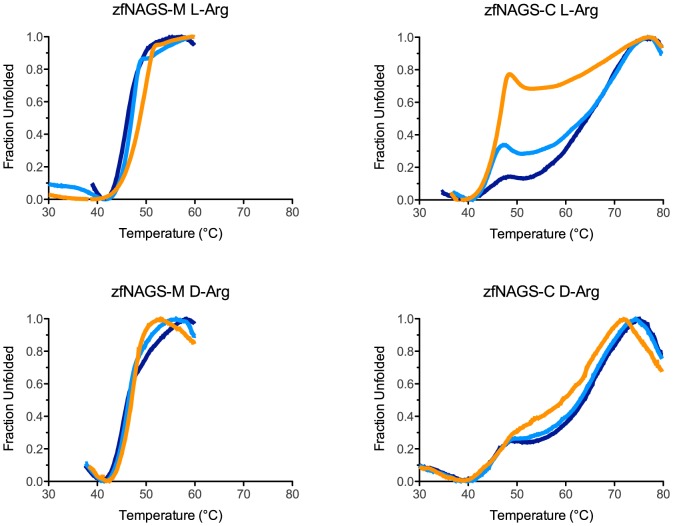
Thermofluor® analysis of zebrafish NAGS in the presence and absence of L- and D-arginine. Unfolding of zfNAGS-M was measured in the presence of increasing concentrations of either L-arginine (A) or D-arginine (B). Unfolding of zfNAGS-C was measured in the presence of increasing concentrations of either L-arginine (C) or D-arginine (D). Dark blue – thermal unfolding in the absence of L- or D-arginine. Cyan - thermal unfolding in the presence of 1 mM L- or D-arginine. Orange - thermal unfolding in the presence of 10 mM L- or D-arginine.

Both zfNAGS-M and zfNAGS-C require acetone, imidazole and TritonX-100 [3] to be soluble and even with these additives both proteins aggregate at concentrations above 1.3 and 2.0 mg/ml, respectively. This prevented the use of biophysical methods such as circular dichroism [81], [82], isothermal titration calorimetry [83] and tryptophan fluorescence measurements [84] to determine effects of either the variable segment or L-arginine on stability of zebrafish NAGS. Thermofluor® is a method that relies on changes in fluorescence of environment-sensitive dyes to track thermal unfolding of proteins in the presence or absence of ligands [85]. We used SYPRO Orange to track the unfolding of zfNAGS-M and zfNAGS-C with or without L- or D-arginine (Figure 7). Both zfNAGS-M and zfNAGS-C had multi-state unfolding transition curves (Figure 7), which was expected, as monomers of both proteins have two structural domains and both proteins are oligomers. The thermal denaturation behaviors of zfNAGS-M and zfNAGS-C were different (Figures 7A and C) suggesting that removal of the variable segment results in different ensembles of molecules each with its own set of unfolding trajectories. The transition from folded to unfolded state occurred over 20°C for zfNAGS-M (Figure 7A) whereas unfolding of zfNAGS-C occurred over 40°C (Figure 7C) suggesting that zfNAGS-C might be a more diverse ensemble of molecules that, as a group, unfold over broader temperature range than zfNAGS-M. Because both unfolding and analytical gel chromatography experiments suggest that zfNAGS-C may exist as a broader ensemble of molecules, we speculate that the variable segment in zfNAGS-M functions to stabilize zebrafish NAGS oligomerization state.

Addition of L-arginine to both zfNAGS-M and zfNAGS-C resulted in changes of the shape of unfolding curves (Figures 7A and C) suggesting that binding of L-arginine induces conformational changes that make hydrophobic regions of both proteins accessible to SYPRO Orange and result in increased fluorescence. Large changes in fluorescence intensity were absent when D-arginine was added to zfNAGS-M and zfNAGS-C (Figures 7B and D) indicating that fluorescence changes in Figures 7A and 5C were not due to interaction between SYPRO Orange and arginine. Addition of 1 and 10 mM L-arginine to zfNAGS-M resulted in its stabilization by 1° and 3°C, respectively (Figure 7A). This indicates that L-arginine can bind to zfNAGS-M when zebrafish NAGS substrates are absent.

### Summary

Zebrafish NAGS, the five urea cycle genes and two transporters are expressed during the first four days of development, a time when neurogenesis takes place [49] and before gills are fully formed [58]. This expression pattern is consistent with excretion of urea after the first day of development but cannot explain ureotely of zebrafish embryos during first 24 hpf [25], [67] because of the absence of Arg1 mRNA. Fish NAGS sequences, like their mammalian homologs, have three distinct regions of sequence conservation including MTS, variable segment and conserved domain, which harbors the catalytic domain and the binding site for the allosteric regulator L-arginine [3], [41]. Upon binding of L-arginine both zfNAGS-M and zfNAGS-C exhibit pronounced change in oligomerization and their enzymatic activity is reduced by 30–50%. In the presence of L-arginine the apparent V_max_ values of both zfNAGS-M and zfNAGS-C decreases and the K_m_^app^ for AcCoA increases while the K_m_^app^ for glutamate remains unchanged. Compared to the estimated physiological concentrations of AcCoA and glutamate [75], [76], the values of K_m_^app^ in the presence of L-arginine suggest that zebrafish NAGS catalyzes the formation of NAG at a maximal rate and that the rate of ureagenesis in zebrafish likely depends on the concentration of urea cycle intermediates.

## Methods

### Ethics Statement

Experimental procedures involving developing zebrafish were approved by the Institutional Animal Care and Use Committee of the Rowan University. The protocol number was 2010-001.

### Purification and real-time quantification of mRNA

Zebrafish embryos from the following nine developmental stages were collected and flash frozen in liquid nitrogen: 32 cells, 30% epiboly (4.6 hpf), 90% epiboly (9 hpf), tailbud (10 hpf), 24 hpf, 48 hpf, 72 hpf, 96 hpf, 105 hpf. Two adult fish were euthanized with tricaine [86] during daytime and flash frozen in liquid nitrogen. Between 100 and 150 embryos and larvae were used for RNA purification. Total RNA was purified using trizol reagent (Invitrogen). One µg of purified RNA was reverse transcribed into cDNA using random primers and SuperScriptIII Reverse Transcriptase kit (Invitrogen) according to manufacturers instructions. The cDNA was used as a template for quantitative real-time PCR using iTaq SYBR Green Supermix with ROX (Bio-Rad) with an ABI 7900HT Sequence Detection System (Applied Biosystems) and primers listed in Table 3. Amplification products were subjected to thermal melting curve analysis to exclude non-specific products and primer-dimer formation. Unlike mammals, zebrafish genome has only one copy of the ASL gene and one citrin/Aralar gene.

**Table 3:** Primers that were used for quantitative RT-PCR of urea cycle genes in the developing zebrafish.

Gene	Primer Sequence
**NAGS**	5′-AGCATCTCTGGAGGGCAGACTGCATTCT-3′
	5′-GGAGTCAGGATGGGACTTGGCGAACTC-3′
**CPS3**	5′-TTGCCTGGCCGAGCGTTGAAACC-3′
	5′-TTGGCGGTAGTGGAACAGGC-3′
**OTC**	5′-TTGCACATTTCAAAGGTTATGAGCCAGATG-3′
	5′-ACCCATAATGGTCCACTTGCGGTTCTC-3′
**ASS**	5′-CTATGGACCGCGAGGTGCGCACG-3′
	5′-CCTTGTAGACGGAGAGCTGGACTCG-3′
**ASL**	5′-GACACTCAAAGGCTTACCAAGCACGTACAAC-3′
	5′-CCAGCATATCTGGACTGAGGGCTTCTTC-3′
**Arg1**	5′-AGTTTCGACATTGATGCGCTGGAC-3′
	5′-CCAGTTTGGGGTTCACTTCCACC-3′
**OTNT**	5′-TTTGACCACAACCATTGCCCGTGAG-3′
	5′-GTCCGAATCATAGTGGGAGTCAGACCAGAAT-3′
**Aralar**	5′-GCTCGTCTCCTCAGTTCGCTGTGAC-3′
	5′-CGGTAACCACCGACATGCTCAGA-3′

### Cloning and Plasmid Preparation

The N-terminus of zfNAGS-M protein is at the Met^47^ of zebrafish preprotein, which was determined based on the prediction of MTS by the MitoProt software package [73]. The N-terminus of zfNAGS-C is at the Gly^73^ of preprotein sequence, which was determined based on the alignment of zebrafish and mammalian NAGS. The coding sequence of zebrafish NAGS-M and NAGS-C were amplified with primers 5′-CGGCATATGAGCTCTTCCAGCACCGCTGG-3′ and 5′-GAGAGGATCCTTATTATT-ATGAGCCGTGGTGCTGCTGAAGAGG-3′, and 5′-ACTCGCATATGGGTGAGCGCAG-CGCCTGG-3′ and 5′-GAGAGGATCCTTATTATTATGAGCCGTGGTGCTGCTGAAG-AGG-3′, respectively using 10 ng of pET15bzfNAGS [3] as template and the following conditions: 3 min. initial denaturation at 95°C, followed by 25 cycles of 30 s denaturation at 95°C, 30 s annealing at 55°C and 1.5 min extension at 72°C, and 5 min. final extension at 72°C. Amplification products were subcloned into pCR4Blunt-TOPO plasmid (Invitrogen) to generate pTOPOzfNAGS-M and pTOPOzfNAGS-C. These plasmids were cleaved with *Nde*I and *Bam*HI restriction endonucleases and zebrafish NAGS-M and NAGS-C coding sequences were subcloned into pET15b plasmid to yield pET15bzfNAGS-M and pET15bzfNAGS-C, respectively.

### Protein Purification and Enzyme Assays

Recombinant zebrafish NAGS was overexpressed in *E. coli* and purified as described previously [3]. Briefly, expression plasmids were transformed into C41(DE3) *E. coli* cells and overexpression of recombinant proteins was induced using an Overnight Expression Autoinduction System 1 (Novagen). Cells were pelleted and resuspended in buffer A (50 mM potassium phosphate buffer, pH 7.5, 300 mM KCl, 10 mM β-mercaptoethanol (BME), 0.006% TritonX-100, 20% glycerol and 1% acetone) containing 10 mM imidazole. Resuspended cells were treated with lysozyme and phenyl-methylsulfonyl fluoride, and lysed with 40 mM *n*-octyl-β-D-glucopyranoside. Nucleic acids were removed with DNase1 and RNaseA. Cleared cell lysates were loaded onto HisTrap™ HP Ni-affinity column (Amersham Biosciences) that was pre-equilibrated with buffer A. The column was sequentially washed with buffer A containing 50, 125, 250 and 500 mM imidazole. Recombinant zfNAGS proteins eluted between 125 and 500 mM imidazole. Elution fractions with 250 mM imidazole were used for experiments. Purified zfNAGS-M and zfNAGS-C could not be concentrated above approximately 1.3 mg/ml and 2.0 mg/ml, respectively, because they aggregated and precipitated.

Enzymatic activities of purified proteins were measured, as described previously [87], with minor modifications. Substrate concentrations in the assays for kinetic measurements were one of the following: 4 mM AcCoA while varying L-glutamate between 0.5 and 10 mM, or 15 mM L-glutamate while varying AcCoA between 0.125 and 2.5 mM. Each assay was performed in triplicate with 8 µg/ml of enzyme. Where indicated, 0.2, 0.5 and 2 mM arginine was added. The data were fit to the Michaelis–Menten equation to determine K_m_^app^ and k_cat_, and to a hyperbolic function to determine K_i_ using GraphPad Prism 5.0 software and non-linear least squares regression.

### *Analytical Gel Chromatography*

Analytical gel chromatography experiments were performed at room temperature. A Superdex 200 HR 10/30 column (Amersham) was calibrated with a buffer that contained 50 mM potassium phosphate pH 7.5, 300 mM KCl, 20% glycerol, 10 mM β-mercaptoethanol, and 0.006% Triton X-100 with and without 1 mM L-arginine at a constant flow rate of 0.75 ml/min using a Pharmacia Acta FPLC system. The column was calibrated with ferritin, catalase, aldolase, bovine serum albumin, ovalbumin, and myoglobin. Void and internal volume markers were blue dextran and vitamin B12. Protein concentrations of recombinant zebrafish NAGS were measured using Bradford assay (Biorad) and bovine serum albumin as a standard. 100 µl of zfNAGS, at concentrations indicated in the figures, was loaded onto the column. To ensure that NAGS integrity was not affected by chromatography, the enzymatic activity of the recombinant NAGS was measured before loading onto the column and after elution. Elution fractions containing NAGS were pooled and used for determination of the total enzymatic activity, which was similar to the total activity of loaded protein.

### Thermal Stability and Ligand Binding

Thermal stability assays were performed in a 96 well plate format using a 7900HT Real-Time PCR System (Applied Biosystems). Protein unfolding was monitored by measuring the change in fluorescence intensity of SYPRO Orange (Invitrogen) while ramping temperature from 4°C to 99°C. Wells contained 10 µg of enzyme in 50 mM potassium phosphate pH 7.5, 300 mM KCl, 20% glycerol, 250 mM imidazole, 10 mM BME, 0.006% Triton X-100, 1% acetone, and 20x SYPRO Orange. Where indicated 10 mM L- and D-arginine were added to the assay mixture. All measurements were carried out in triplicate.

## Supporting Information

Figure S1 **Dependence of the rate of reaction catalyzed by zebrafish NAGS proteins on the concentrations of AcCoA and glutamate.** When AcCoA concentration was varied glutamate concentration was fixed at 15 mM. When glutamate concentration was varied AcCoA concentration was fixed at 4 mM. The assays were performed in the absence (dark blue), 0,2 mM (orange), 0,5 mM (green) or 1 mM (red) L-arginine. The curves were fitted to Michaelis–Menten equation using GraphPad Prism 5.0 software.
(DOCX)
