# Supplementary material for: Expression Pattern and Biochemical Properties of Zebrafish N-Acetylglutamate Synthase
Source: PLoS One. 2014 Jan 22;9(1):e85597. doi: 10.1371/journal.pone.0085597 (PMC3899043; doi:10.1371/journal.pone.0085597)
Supplement: Figure S1 — Dependence of the rate of reaction catalyzed by zebrafish NAGS proteins on the concentrations of AcCoA and glutamate. When AcCoA concentration was varied glutamate concentration was fixed at 15 mM. When glutamate concentration was varied AcCoA concentration was fixed at 4 mM. The assays were performed in the absence (dark blue), 0,2 mM (orange), 0,5 mM (green) or 1 mM (red) L-arginine. The curves were fitted to Michaelis–Menten equation using GraphPad Prism 5.0 software. (DOCX) [file pone.0085597.s001.docx]

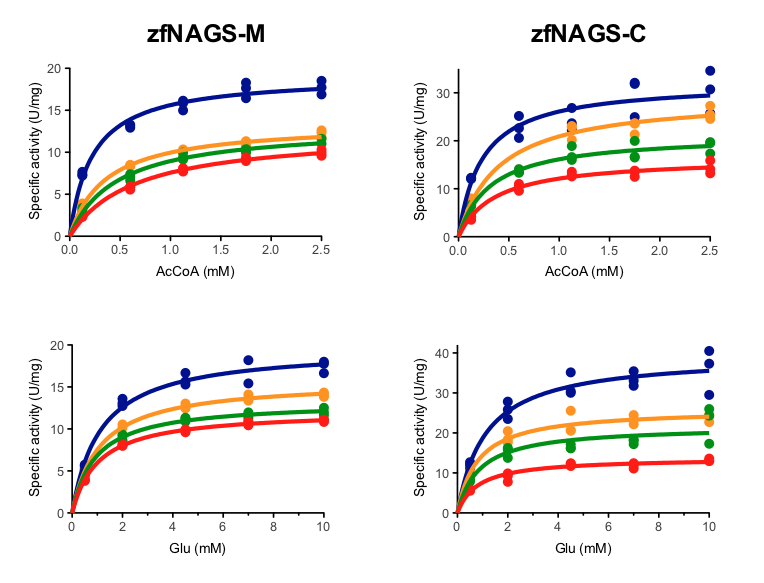


**Figure S1.** Dependence of the rate of reaction catalyzed by zebrafish NAGS proteins on the concentrations of AcCoA and glutamate. When AcCoA concentration was varied glutamate concentration was fixed at 15 mM. When glutamate concentration was varied AcCoA concentration was fixed at 4 mM. The assays were performed in the absence (dark blue), 0,2 mM (orange), 0,5 mM (green) or 1 mM (red) L-arginine. The curves were fitted to Michaelis–Menten equation using GraphPad Prism 5.0 software.
